# Transparent Conductors Printed from Grids of Highly Conductive Silver Nanosheets

**DOI:** 10.1021/acsami.3c07459

**Published:** 2023-08-10

**Authors:** Adam G. Kelly, Siadhbh Sheil, Danielle A. Douglas-Henry, Eoin Caffrey, Cian Gabbett, Luke Doolan, Valeria Nicolosi, Jonathan N. Coleman

**Affiliations:** † School of Physics, CRANN and AMBER Research Centres, Trinity College Dublin, Dublin D2, Ireland; ‡ School of Chemistry, CRANN and AMBER Research Centres, Trinity College Dublin, Dublin D2, Ireland

**Keywords:** transparent conductors, printed electronics, silver nanoplatelets, optoelectronics, conductive inks, flexible electronics, solution processing

## Abstract

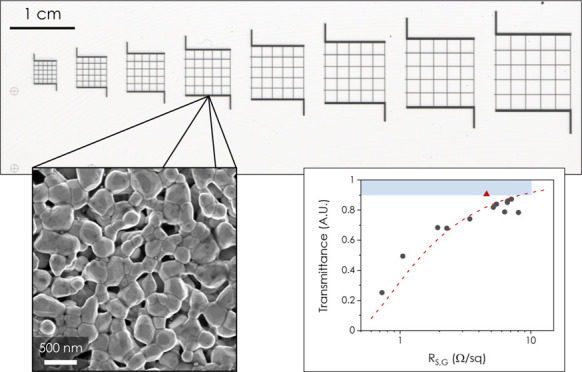

Transparent conductors (TCs) represent key components in many applications from optoelectronic devices to electromagnetic shielding. While commercial applications typically use thin films of indium tin oxide, this material is brittle and increasingly scarce, meaning higher performing and cheaper alternatives are sought after. Solution-processible metals would be ideal owing to their high conductivities and printability. However, due to their opacity to visible light, such films need to be very thin to achieve transparency, thus limiting the minimum resistance achievable. One solution is to print metallic particles in a grid structure, which has the advantages of high tunable transparency and resistance at the cost of uniformity. Here, we report silver nanosheets that have been aerosol jet printed into grids as high-performance transparent conductors. We first investigate the effect of annealing on the silver nanosheets where we observe the onset of junction sintering at 160 °C after which the silver network becomes continuous. We then investigate the effect of line width and thickness on the electrical performance and the effect of varying the aperture dimensions on the optical performance. Using these data, we develop simple models, which allow us to optimize the grid and demonstrate a printed transparent conductor with a transmittance of 91% at a sheet resistance of 4.6 Ω/sq.

## Introduction

Transparent conductors (TCs) are critical materials in the field of printed electronics where they constitute core components of solar cells,^1^ light emitting diodes (LEDs),^2^ and touch panels.^3^ Two important, but inversely desired, characteristics of transparent conductors are their transmittance (*T*) and sheet resistance (*R*_S_), where a high-performance thin film should display a *T* ≥ 90% with an *R*_S_ ≤ 10 Ω/sq.^4^ Such properties generally require the maximization of the DC conductivity of the thin film (more specifically the ratio of DC to optical conductivity).^4^ Until recently, continuous layers of metal oxides, primarily indium tin oxide (ITO), were commonly used in commercial applications owing to their high transparency at low film thickness and sufficiently high conductivity leading to *T* ≥ 90% and *R*_S_ ≤ 10 Ω/sq for most types of ITO. However, these materials are often brittle, expensive, and scarce,^5^ which has led to the exploration of a number of alternative materials and deposition methods to address these shortcomings.

For device applications, it must be possible to deposit transparent conductive thin films with areas of ∼cm^2^. To achieve this, processing in solution and deposition by printing is advantageous due to its speed, scalability, and low cost and much work has been done on a range of printable transparent conductors. Conductive polymers such as PEDOT:PSS have been investigated due to their solution processibility and high transmittance when printed into a thin film. Although as-deposited conductivities are quite low (∼1 S m^–1^), post-processing techniques can raise this by several orders of magnitude to ∼10^4^ S m^–1^ [ref (5)]. The most effective method of increasing the conductivity of PEDOT:PSS is treatment using strong acids,^6^ which renders this process incompatible with flexible substrates such as PET while the material itself also has low transmittance at higher wavelengths.^1^ Chemical post-treatments can be avoided by using carbonaceous nanomaterials such as graphene or carbon nanotubes, which have also been explored as printed transparent conductors.^5^ While both of these materials can achieve a transmittance >90% in thin films (typically <50 nm), conduction in printed networks of both materials is almost always suppressed by the inter-sheet/inter-tube junctions, which limits the conductivity to the range of 10^2^–10^4^ S m^–1^ [ref (7)]. To achieve low sheet resistances therefore requires thick networks to be printed, which naturally leads to a reduction in transmittance meaning carbon-based TCs generally struggle to achieve *R*_S_ ≤ 100 Ω/sq for films with *T* = 90%.

To achieve low sheet resistances, networks of various metallic nanowires such as gold (AuNW),^8^ silver (AgNW),^9,10^ and copper (CuNW)^11^ have been demonstrated as transparent conductors with each showing similar or superior performance to ITO thin films. The high porosity associated with a nanowire network (>90%)^12^ confers a high transmittance, and the metallic conductivity (>10^6^ S/m is possible)^10^ allows low sheet resistances to be achieved. However, the as-deposited conductivity of the networks can be low owing to large junction resistances where the nanowires come into contact within the network unless annealing is performed.^9^ In addition, the porosity of the nanowire network can make it difficult to integrate with active layers. The nanowires themselves can also penetrate active layers in devices such as solar cells, which leads to reduced device performance or, in the worst case, an electrical short.^1^

Other metallic nanomaterials such as nanoparticles of silver (AgNP)^13^ or copper (CuNP)^14^ have also been investigated as transparent conductors but tend to form densely packed networks with low porosity meaning they appear opaque and highly reflective to visible light. This requires a grid design to be printed where the grid lines are composed of the metallic nanomaterial leaving the apertures in between completely transparent to incident light.^13,15^ In such a structure, *T* is controlled by the ratio of aperture width-to-line width, while *R*_S_ is controlled by line dimensions and the conductivity of the material. As AgNPs or CuNPs often have diameters in the range of 20–80 nm and are roughly spherical in shape, the inter-particle interface is small, which leads to poor inter-particle charge conduction and low as-printed conductivities. This means that thermal post-treatments are required to sinter the particles and lower the sheet resistance of the network, a process that again is usually incompatible with flexible substrates. The sheet resistance of the grid can also be lowered by coating the grid in a continuous network of CNTs,^16^ PEDOT:PSS,^17^ or even ITO.^18^ While such coatings will clearly lower the transmittance, they often result in only modest reductions in sheet resistance meaning the added processing complexity and decreased transmittance may outweigh the potential electrical improvements.

As all of the printed thin films discussed so far are composed of discrete components, achieving a high conductivity (i.e., low *R*_S_) is usually limited by the junctions between the particles.^7^ In AgNW and AgNP networks, the junction is size-limited by the diameter of the wires/particles, which in turn limits the minimum achievable junction resistance. For example, AgNW networks can have junction resistances of tens of Ohms following annealing.^19^ However, in recent work,^20^ we reported silver nanosheets (AgNS) as a printable conductor with a high as-printed conductivity owing to their two-dimensional (2D)-like geometry with large basal planes leading to low junction resistances of ∼3 Ω. In addition, they form films with smooth surfaces and interfaces, which makes them ideal for preventing electrical shorting in vertical heterostructures composed of porous networks. The 2D-like geometry is also highly beneficial for creating networks on flexible substrates as they are more robust against crack formation compared to silver nanoparticles under bending tests.^21^

Here, we exploit these properties to explore and characterize their utility in printed grids that can be used as transparent conductors. We first investigate the effect annealing has on the AgNS using transmission electron microscopy (TEM), scanning electron microscopy (SEM), and electrical measurements. We then evaluate the way in which the dimensions of the printed lines affect the sheet resistance of the grid. We vary the aperture size to characterize its effect on both transmittance and grid sheet resistance. With these parameters known, we can then print a grid that performs with a *T* = 91% with an *R*_S_ = 4.6 Ω/sq.

## Results and Discussion

We initially printed grids with a range of thicknesses and aperture sizes to act as transparent conductors using water-based AgNS inks with a commercial inkjet printer following on from our previous work.^20^ These grids displayed an as-printed conductivity of ∼7.5 × 10^6^ S m^–1^, higher than all reported as-printed AgNW networks, and *R*_S_ in the range of 2–5 Ω/sq (see Supporting Information S1). However due to the resolution limits of this printer, there is a lot of scatter in the data and the minimum line width is ∼500 mm [ref (20)], meaning apertures greater than 10 mm are necessary to achieve transmittances >90%. We therefore moved to a high-resolution aerosol jet printer^22^ for the rest of this work.

**Figure 1:**
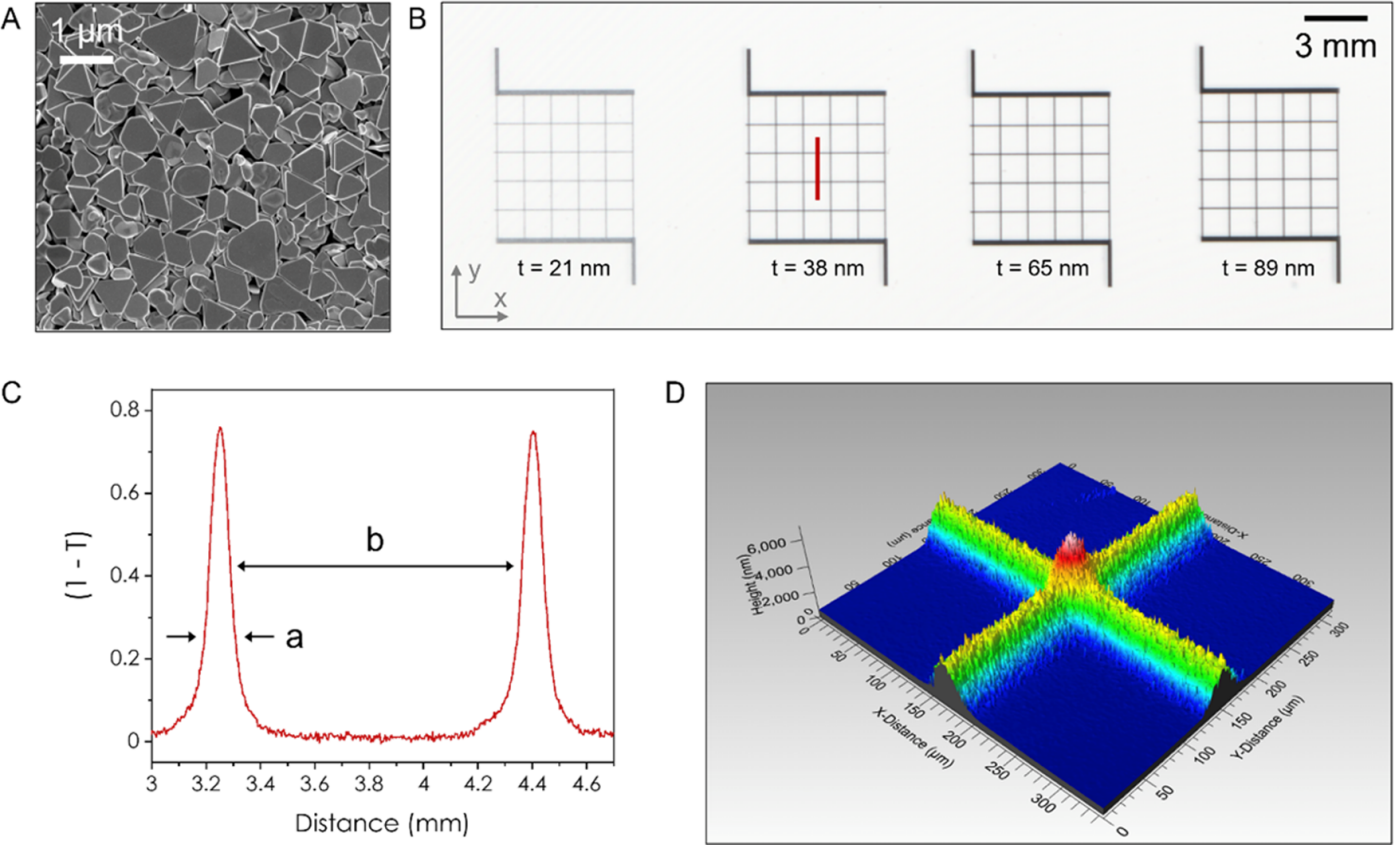
Material and Grid Characterization. (A) SEM image of the AgNS. (B) Optical image obtained by a transmission scanner of four grids with the grid thickness given underneath. The red line on the 38 nm grid indicates where the profile in (C) was extracted from. (C) Plot of the central lines shown in panel (B), where *a* indicates the line width and *b* indicates the aperture size. Such graphs allow the determination of *a* and *b* by fitting the line width to a gaussian to get the FWHM. (D) White-light interferometry on a thick grid (*t* ∼ 2.4 μm), which allows accurate thickness measurements and optical characterization.

Figure 1A shows an SEM image of the dropcast AgNS where they are visibly polydisperse with lengths of 300–1000 nm and an average thickness of 45 nm.^20^ An ink suitable for aerosol jet printing based on a 90:10 blend of cyclohexanone and terpineol (C/T) was prepared following the procedures reported in ref (20). Grids with a range of thicknesses were printed onto a transparent glass slide, and images were taken using a transmission scanner to allow measurement of characteristics such as line width, *a*, aperture size, *b*, and the transmittance of the grid, *T*. Figure 1B shows four grids with different thicknesses (*t*) while holding *a* and *b* constant. We plot 1 – *T* (i.e., the sum of the absorbance and reflectance of the AgNS) against position then allows one to estimate the shape of the lines making up the grid. Figure 1C shows a distance profile for the second grid in Figure 1B (indicated by the red line) allowing the extraction of the FWHM of the printed line (as an estimate of the line width, *a*) and also peak-to-peak distance (from which the line width is subtracted to give the aperture size, *b*) as demonstrated in SI S2. Finally, the thickness of the grids can be measured using white-light interferometry (WLI) producing maps such as that shown in Figure 1D. This technique is a highly valuable method of obtaining accurate film thicknesses as it is both non-contact and substrate-agnostic (see Experimental Methods), which allows a rapid, accurate, and non-destructive estimation of the thickness of the printed features.

**Figure 2:**
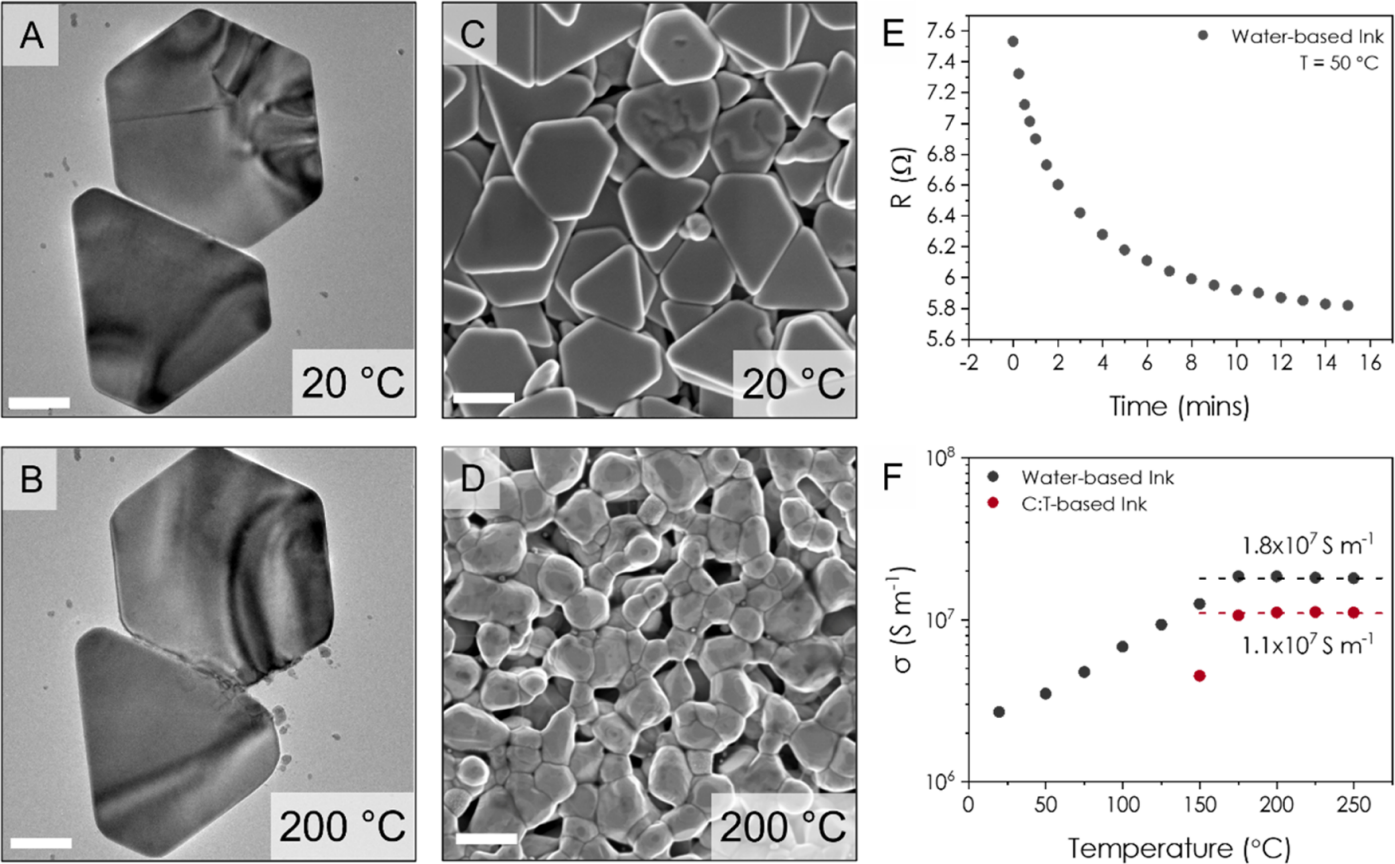
Effect of Annealing. (A) TEM image showing two AgNS in contact at room temperature. Scale bar = 100 nm. (B) Same AgNS following annealing at 200 °C where sintering along the contact edge can be seen. Scale bar = 100 nm. (C) SEM image of inkjet-printed AgNS using a water-based ink. Scale bar = 500 nm. (D) Same network following annealing at 200 °C where the nanosheets have visibly sintered together into a continuous network. Scale bar = 1000 nm. (E) Change in resistance of an inkjet-printed AgNS network (a continuous film, not a grid) over time while the network is being heated at 50 °C. The decay in resistance saturates after approximately 15 min. (F) Evolution of conductivity with annealing temperature. The networks were annealed at the indicated temperature for 15 min and allowed to cool to room temperature before measurement. The inkjet-printed water-based ink is conductive at all temperatures and saturates at ∼1.8 × 10^7^ S m^–1^ between 150 and 175 °C. This compares to the aerosol jet printed C/T-based ink, which only shows conduction following annealing at 150 °C but also saturates at 175 °C with a conductivity of ∼1.1 × 10^7^ S m^–1^. We attribute the difference in saturated conductivity to the residual presence of the high boiling point solvents used in the C/T ink.

Figure 2 shows the effect of annealing the AgNS. In AgNW networks, filaments are known to form across the junctions with increased annealing temperature, which leads to the increase in conductivity of the network. To investigate the effect of annealing on AgNS networks, we used in situ heating transmission electron microscopy to capture the sintering at the junctions with thermal treatment. Figure 2A shows a TEM image of two AgNS lying edge on at room temperature. Once a critical temperature is reached, the junctions weld together in a zipper-like effect as captured in Supporting Video 1 and shown in SI S3. The fused edges then create a large sintered junction with an area of ∼13,500 nm^2^ for the junction in Figure 2B, which compares to a junction area of ∼2000 nm^2^ for AgNW with a diameter of 50 nm. Figure 2C,D shows SEM images of a printed AgNS network (i.e., not a grid) before and after annealing in ambient conditions, where the entire structure of the network changes once the AgNS reach 200 °C. While the large basal plane interfaces shown in Figure 2C allow room temperature network conductivities up to 3 × 10^6^ S m^–1^ to be reached, once the network is annealed above 150 °C, the AgNS become a continuous sintered network. Furthermore, we also observe the formation of filaments across the pores in the network where they can reach lengths up to 250 nm (SI S4).

To evaluate the optimal annealing conditions, we compared a water-based ink (inkjet printed on a paper substrate) and a C/T-based ink (aerosol jet printed onto glass). Figure 2E shows the 4-probe resistance decrease over time for the inkjet-printed network heated continuously at 50 °C where we find an exponential decay in resistance until it reaches a plateau after approx. 15 min. Figure 2F shows the relationship between the 4-probe conductivity and annealing temperature for both inks. The network conductivity of the water-based ink rises continuously from room temperature until it reaches a plateau of 1.8 × 10^7^ S m^–1^ at 175 °C. This coincides with the onset of junction sintering shown in SI S3. However, the C/T ink is non-conductive until 150 °C owing to the large amount of residual high-boiling-point solvent remaining in the network. Following the onset of conduction, this ink plateaus at 1.1 × 10^7^ S m^–1^ at 175 °C as the sintering of the junctions negates the effect of residual solvent. The lower value at saturation may be caused by the unevaporated high-boiling-point solvents remaining in the network. This compares to AgNW networks where the conductivity maximum occurs between 225–300 °C depending on the diameter of the nanowire.^9,23^ We note that these networks are measured and stored in ambient where, absent any encapsulation, we expect the conductivity to fall to approximately 66% of these values after a period of one year.^20^ For the rest of this work, we print a C/T ink using an aerosol jet printer to create grids, which are annealed at 200 °C for 15 min following printing.

**Figure 3:**
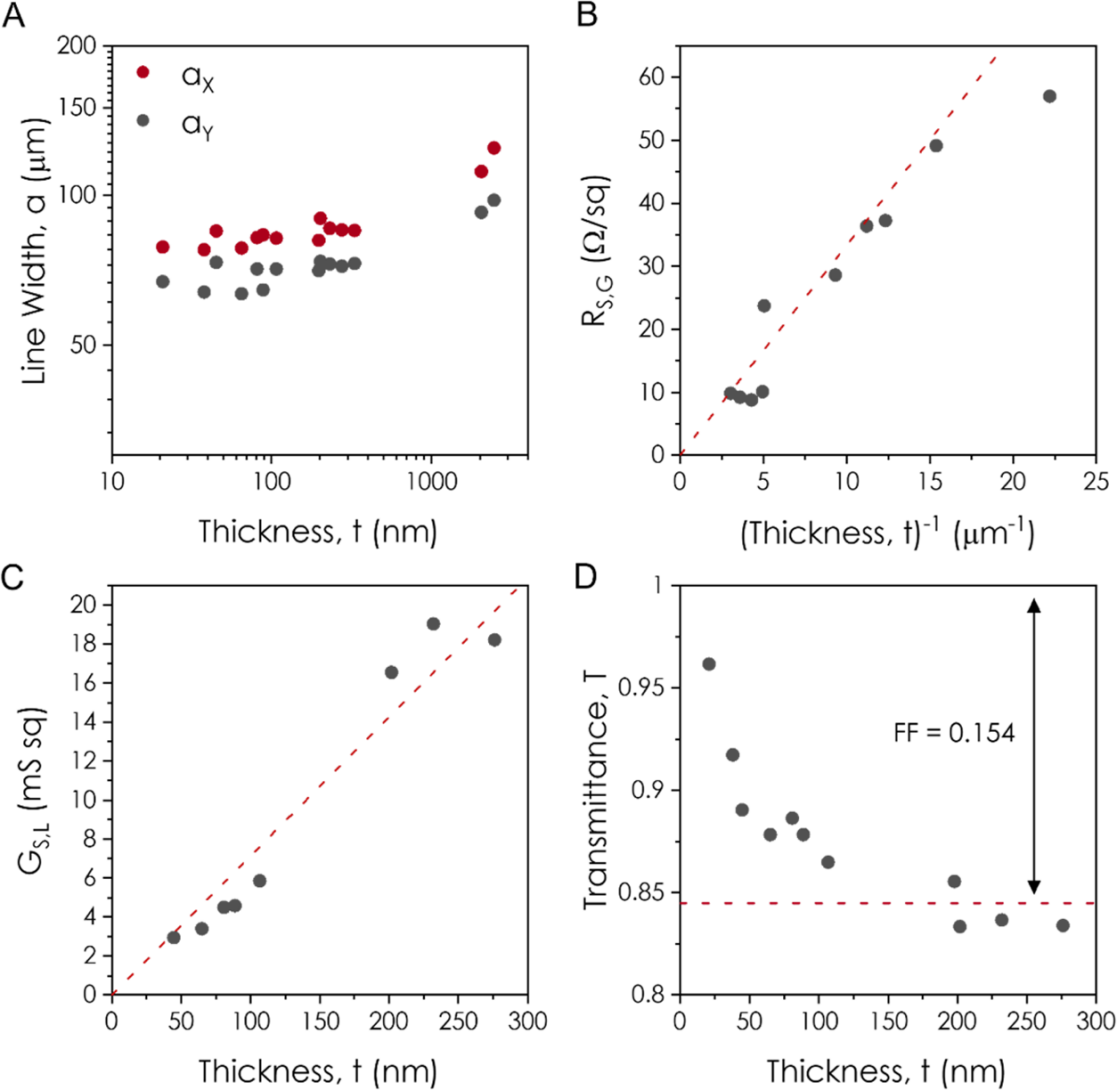
Effect of line thickness. (A) Line width, *a*, plotted against line thickness, *t*, for a range of thicknesses. The line width measured in the *x*-direction (coordinates in Figure 1B) is slightly wider compared to the line width measured in the *y*-direction. This is caused by non-circular deposition by the aerosol jet printer, which occurs when small pieces of debris become lodged in the nozzle. For higher thicknesses, the line width tends to increase, which we attribute to residual solvent on the substrate causing the material to redisperse and flow. (B) Sheet resistance of the grid, *R*_S,G_, plotted against the inverse thickness. The dashed line is a fit to *R*_S,G_ = (σ_G_*t*)^−1^. (C) Conductance of a single line plotted against the line thickness. The red dashed line is a fit to *G*_S,L_ = *A*_L_*σ_L_/*L* as discussed in the main text. (D) Transmittance of each grid plotted against the grid thickness. This demonstrates that below a certain thickness, the lines have a non-zero transmittance, which contributes to the overall transmittance of the grid. This occurs in the region below the thickness-independent conductivity limit, which occurs at *t* ∼140 nm for AgNS networks.^20^

To investigate the effect of line thickness on the structural, optical, and electrical characteristics of the grids, *a* and *b*, were set to be constant at *a* = 85 μm and *b* = 1.15 mm while successive layers were printed. These parameters are controlled by the settings on the aerosol jet printer, where *a* is determined by the sheath and carrier flow rates while *b* is set by the toolpath where it indicates the center-to-center distance of the grid lines. Figure 3A shows the change in line width, *a*, with thickness where we see it remain approximately constant up to thicknesses of 350 nm. For the thicker grids, we see a broadening of the line width, which we attribute to solvent accumulation that can occur when using high-boiling-point solvents and a large number of layers. We find that the line width in the *x*-direction is (85 ± 4) μm but (69 ± 4) μm in the *y*-direction. This means the columnar deposition by the aerosol jet is not perfectly circular, which can occur when material becomes lodged inside the nozzle.

The dependence of the 2-probe grid sheet resistance, *R*_S,G_, on the inverse thickness, *t*^–1^, is shown in Figure 3B where the data begin to move away from linearity as the thickness moves below the thickness-independent conductivity limit. The dashed line represents a fit to *R*_S,G_ = (σ_G_*t*)^−1^, which gives a grid conductivity (including the effect of contact resistance), σ_G_, of 3 × 10^5^ S m^–1^, approximately 30× lower than that of an individual line (∼7.5 × 10^6^ S m^–1^).^20^ In Figure 3C, we took the 2-probe sheet conductance of a single line, *G*_S,L_ (= 6/*R*_S,G_, with the factor of six to account for the six lines in each grid, assuming each are independent parallel conductors), and plotted it against the thickness. These data were then fit to *G*_S,L_ = *t*(a*σ_L_)/*L*, where σ_L_ is the conductivity of the line, and *L* is the length of the line. With *L* = 5.6 mm, this gives a σ_L_ of 4.7 × 10^6^ S m^–1^. This value is ∼2.5× lower than that reported in ref (20) as these measurements include a contribution from the contact resistance, which is associated with the combination silver paint bonding agent and the small tag (top right and bottom left of grids in Figure 1B) used to connect to the grid. Here, the contact resistance was not easily measurable and so could not be corrected for to obtain the exact conductivity (discussed further below). However, the real line conductivity is clearly 4.7 × 10^6^ S m^–1^ and may approach the network conductivity of >10^7^ S m^–1^ mentioned above. Figure 3D shows the relationship between grid thickness and transmittance. For thicknesses below the thickness-independent conductivity limit, we find the transmittance trends toward 1 as the grids get thinner as is visible in Figure 1B. Once the line thickness is above the thickness-independent limit of 140 nm (ref (20)), the lines become fully reflective and the transmittance becomes constant with thickness at an average transmittance of 0.84. This is consistent with a calculated fill factor (FF), the proportion of the grid occupied by the printed lines, of 0.154 (see SI S5).

**Figure 4:**
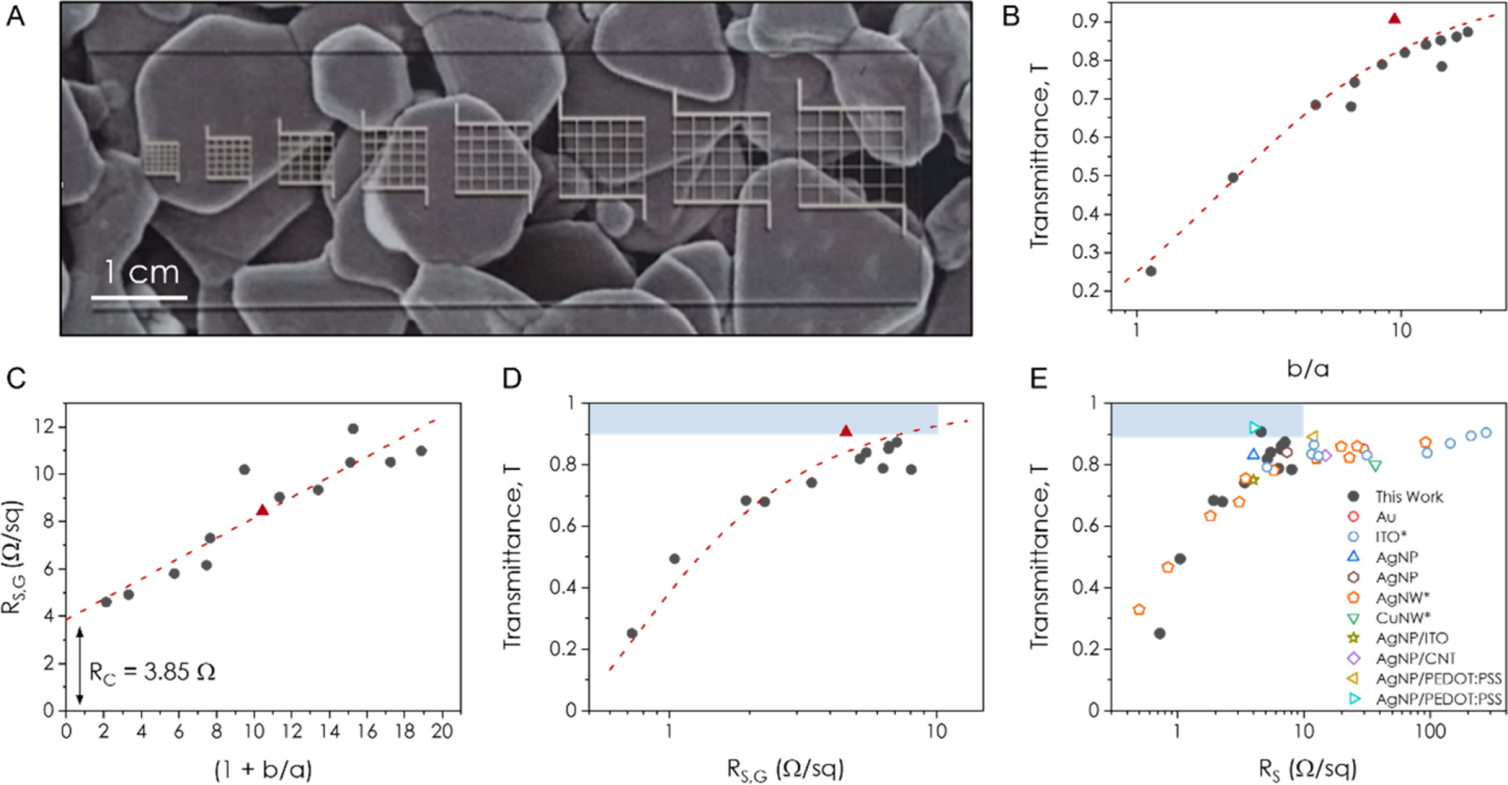
Effect of Aperture Size. (A) Photo of 8 grids of varying aperture size printed onto a microscope slide. The microscope slide is placed upon a printed-out SEM image of the AgNS network. (B) Variation of transmittance with aperture size, with line width held constant at ∼100 μm. The red triangle indicates a grid printed using the optimal conditions to create a high-performance transparent conductor while the dashed line is a plot of eq 1. (C) Relationship between the sheet resistance of the grid and aperture size, with line width again held constant at ∼100 μm. The intercept at (1 + *b*/*a*) = 0 indicates the contact resistance. The red triangle indicates a grid printed using the optimal conditions to create a high-performance transparent conductor and the fit is to eq 2. (D) Dependence of transmittance on the grid sheet resistance following the correction for contact resistance. The dashed line is a fit to eq 3, the red triangle is again the optimally printed grid, and the blue box indicates the region of the graph with *T* ≥ 90% and *R*_S,G_ ≤ 10 Ω/sq. (E) Literature review of a range of grids fabricated from different nanomaterials where the materials marked with an asterisk represent continuous networks while all others are grids. Data taken from refs (10, 16−18, 24−28). The blue box again indicates the region of the graph with *T* ≥ 90% and *R*_S,G_ ≤ 10 Ω/sq.

With the effect of grid thickness determined, we then investigated the effect of varying the aperture size while maintaining constant thickness (*t* ∼ 200 nm) and line width (*a* ∼ 100 μm). Figure 4A shows an image of 8 grids with a range of aperture sizes printed on a microscope slide. The slide was then placed on top of an SEM image of the AgNS to demonstrate the grid transparency. From the SI S5, assuming the AgNS lines are fully reflective, the transmittance of a grid is given by
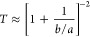
1where *a* is the line width and *b* is the aperture size. Figure 4B shows the transmittance for several such grids plotted against *b*/*a*, with *a* fixed at 100 μm. We find the data to be in perfect agreement with eq 1.

The relationship between the grid sheet resistance, *R*_S,G_, and *b*/*a* is shown in Figure 4C where we see linear behavior including an offset at *b*/*a* = 0. The 3.85 Ω offset is due to the effect of contact resistance due to the silver paint and contact tag as mentioned above. As shown in SI S6, the relationship between *R*_S,G_ and *b*/*a* is given by
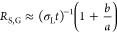
2where σ_L_ and *t* are the conductivity and thickness of an individual line, respectively, and the inverse product, (σ_L_*t*)^−1^, represents the sheet resistance of a single line, *R*_S,L_. The fit indicated by the dashed line in Figure 4C gives (σ_L_*t*)^−1^ = 0.43 Ω/sq implying a conductivity of σ_L_ = 1.2 × 10^7^ S m^–1^ for *t* = 200 nm (consistent with the data in Figure 2 and that in our previous report^20^).

To evaluate the relationship between *T* and *R*_S,G_, Figure 4D shows the transmittance for each grid plotted against its sheet resistance (where *R*_S,G_ has been corrected for contact resistance by subtracting 3.85 Ω in all cases, see Figure 4C). The best performing grid has values of *R*_S,G_ = 7.12 Ω and *T* = 87%, which are extremely promising compared to most of the literature. The data are well fit by combining eqs 1 and 2 (see SI S7) into
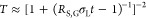
3which gives a fit value of (σ_L_*t*)^−1^ = 0.38 Ω/sq. This implies a conductivity of σ_L_ = 1.3 × 10^7^ S m^–1^ in agreement with the value found in Figure 4C. We perform further analysis on the uniformity of these grids in SI S8, where we find the aperture transmittance is highly homogeneous across a given grid for *b*/*a* > 8. However, below this, the aperture-to-aperture variation begins to increase, which we attribute to the line-edge roughness and overspray by the aerosol jet deposition becoming more apparent as size of the aperture approaches that of the line width. We also find that the printed lines are highly uniform along their length with a low coefficient of variation.

It is worth considering how to improve the performance of these grids. Equation 3 means that reducing the sheet resistance of the grid at a given transmittance (e.g., 90% as determined by the grid FF) will only be achievable by increasing the value of σ_L_*t*. As our measured value of σ_L_ = 1.1 × 10^7^ S m^–1^ is already very high, the simplest route is to increase grid thickness, *t*. To demonstrate an improved AgNS-based transparent conductor with characteristics in the region indicated by the blue box in Figure 4D,E, we printed a grid with *a* = 125 μm, *b* = 1.7 mm, and a much-increased thickness of *t* = 2.5 μm. This corresponds to a *b*/*a* of 13.6 which, following the analysis in SI S8, means the transmittance of such a grid is homogeneous across a large area. We found that such a grid does indeed display enhanced performance, *T* = 91% and *R*_S,G_ = 4.6 Ω/sq, and is represented by the red triangle in Figure 4B–D.

Looking at the literature for a range of transparent conductors printed from various materials (Figure 4E), we see a general trend where high transmittances occur at high sheet resistances and vice versa. The materials indicated by an asterisk are continuous networks, while all other materials are grids, or a combination of a grid with a continuous layer of CNTs, PEDOT:PSS, or ITO. We find that the data for printed grids of AgNS are consistent with the trend for continuous networks of AgNW.^10^ The AgNS grids also show a higher transmittance for a given *R*_S_ than AgNPs^13^ even when the AgNP grid has been spin-coated in either PEDOT:PSS^17^ or carbon nanotubes.^16^ This demonstrates that silver nanosheets are excellent candidates for use as transparent conductors in applications such as large-area displays or wearable optoelectronic devices that do not require a high level of uniformity.

## Conclusions

We have demonstrated that silver nanosheets can be printed into grid structures that are suitable for use as transparent conductors. The AgNS are highly conductive immediately following inkjet printing, which allows low sheet resistances to be achieved; however, the low resolution of the printer means that large aperture sizes are necessary to simultaneously achieve a high transmittance. Aerosol jet printing allows high-resolution grids to be printed in the range of 1–10 Ω/sq following corrections to contact resistance. The transmittance of the grid scales with the ratio of aperture size-to-line width, with high transmittance reached at the highest ratios. Using a simple relationship between sheet resistance and transmittance, we determine that printing a grid that lies in the region where *T* ≥ 90% with an *R*_S_ ≤ 10 Ω/sq requires a network ∼2.4 um thick and with a *b*/*a* of 13.5. This work demonstrates that silver nanosheets are highly suitable materials for use in printed transparent conductors.

## Experimental Methods

### Ink Preparation

The silver nanosheets (AgNS) were purchased from Tokusen USA (N300 Nanoflake) and are delivered in a thick water-based paste. From this, 0.5 mL is diluted with 100 mL of deionized water to give a stock dispersion of approx. 80–100 mg mL^–1^ that is confirmed by vacuum filtration and weighing. Once a stock dispersion is made with a known concentration, it was then diluted with DI water to 20 mg mL^–1^, which is suitable for inkjet printing with a Canon Pixma MG2550 thermal inkjet printer.

To create a cyclohexanone/terpineol (90:10) based ink at 20 mg/mL, 5 mL of the stock water-based dispersion (80 mg/mL) is centrifuged at 3700*g* for 120 min and the all of the supernatant is discarded. The sediment is redispersed in a blend of 18 mL of cyclohexanone and 2 mL of terpineol using a vortex mixer and 15 min in a sonic bath. This centrifugation/redispersion step is repeated twice to minimize the amount of water retained in the ink following the solvent exchange.

### Inkjet Printing

Printing using the Canon Pixma MG2550 thermal inkjet printer was performed following the procedures outlined in ref (20). Briefly, the black ink cartridge is opened and thoroughly cleaned out using an ink refill tool before filling with the water-based AgNS ink. The patterns are designed using Powerpoint with all printed features colored in black. The print quality is set to high under print settings. The substrate used was alumina-coated PET.

### Aerosol Jet Printing

In this study, we used an Optomec AJP 300 with a 150 μm nozzle and an ultrasonic atomizer. The sheath and carrier flow rates were set to 90 ± 5 and 17 ± 2 sccm, respectively. The temperature of the chiller used to cool the ink was set to 10 °C and the platen was set to 90 °C. 2 mL of ink was used at a time with the atomizer current set to ∼0.5 A. All printing was performed at platen speeds of 1 mm s^–1^. Prior to use, the substrates were cleaned in KOH and dried with compressed N_2_.

### White-Light Interferometry

The three-dimensional (3D) surface profiles were collected using a Profilm3D Optical Profiler (Filmetrics) operating in white-light interferometry (WLI) mode with a 50× Nikon DI objective lens. For samples <100 nm, phase shift interferometry (PSI) mode was used, which has increased height resolution for thinner profiles. The profiler was calibrated using a gold thin-film on Si/SiO_2_ with a step height of 50 nm, confirmed using AFM. The profiles were levelled (three-point level), and their step heights were determined using ProfilmOnline (Filmetrics) software. Step heights were determined using the histogram step-height setting in ProfilmOnline, which outputs a histogram of the height of each pixel. From this, the baseline height and the top surface of the deposited network were found to calculate the average step height across the area. Once a profile is drawn, it is some smoothed to remove “invalid” pixels using a “remove outliers” function on the ProfilmOnline software. This crops the *z*-axis to remove any stray high or low pixels and then fills in pixels with an average value of the neighboring pixels.

### Electrical Measurements

The conductivity and sheet resistance measurements were measured using a Keithley 2612A sourcemeter controlled by a LabView program. The sourcemeter was connected to the samples using a Suss probe station.

### Transmittance Measurements

The transmittance was measured using flatbed transmission scanner (Epson Perfection V700 Photo) with a bit depth of 48-bits per pixel and a spatial resolution of 6400 dpi. The signal-to-transmittance relationship was found empirically by scanning neutral density filters.

### TEM In Situ Heating

Direct observation of weld formation at elevated temperatures was achieved through in situ transmission electron microscopy (TEM) using a DENS solutions Wildfire TEM holder. AgNS were dropcast onto a DENS solutions Nano-Chip, which consists of a microelectromechanical system (MEMS) based chip design with electron transparent SiN windows. The temperature of the chip is controlled by a 4-point probe. The TEM was subsequently performed on a FEI Titan 80–300 Thermo Fisher Scientific, fitted with a Schottky field emission gun. The operating voltage was set to 300 kV, and images were recorded using a Gatan UltraScan CCD camera. The temperature ramp rate was set to 15 °C/min and heated from RT to 200 °C. Heating was paused for 2 min at 120 °C and at every 10 °C increment after that. Images were captured every 20 s.

## Supporting Information Available

Inkjet-printed transparent conductors (S1); estimation of line width and aperture size (S2); the onset of sintering at AgNS junctions (S3); the formation of filaments across pores (S4); relationship between transmittance and grid dimensions (S5); relationship between sheet resistance and grid dimensions (S6); relationship between transmittance and sheet resistance (S7); grid and line uniformity (S8) (PDF)
Video showing the TEM in situ heating where junction formation can be observed once a critical temperature is reached (Video 1) (AVI)

## Supplementary Material

am3c07459_si_001.pdf

am3c07459_si_002.avi
