# Supplementary material for: Transparent Conductors Printed from Grids of Highly Conductive Silver Nanosheets
Source: ACS Appl Mater Interfaces. 2023 Aug 10;15(33):39864–71. doi: 10.1021/acsami.3c07459 (PMC10450683; doi:10.1021/acsami.3c07459)
Supplement: Supplementary file 1 — am3c07459_si_001.pdf [file am3c07459_si_001.pdf]

Supporting Information for:

Transparent conductors printed from grids of highly conductive silver nanosheets

Adam G. Kelly[1], Siadhbh Sheil[1], Danielle A. Douglas-Henry[2], Eoin Caffrey[1], Cian Gabbett[1], Luke Doolan[1], Valeria Nicolosi[2], and Jonathan N. Coleman[1*]

[1]School of Physics, CRANN and AMBER Research Centres, Trinity College Dublin, Dublin 2, Ireland.
[2]School of Chemistry, CRANN and AMBER Research Centres, Trinity College Dublin, Dublin 2, Ireland.

Corresponding author: *colemaj@tcd.ie

This document contains:

S1: Inkjet-printed transparent conductors

S2: Estimation of line width and aperture size

S3: The onset of sintering at AgNS junctions

S4: The formation of filaments across pores

S5: Relationship between transmittance and grid dimensions

S6: Relationship between sheet resistance and grid dimensions

S7: Relationship between transmittance and sheet resistance

S8: Grid and Line Uniformity

S1: Inkjet-printed transparent conductors

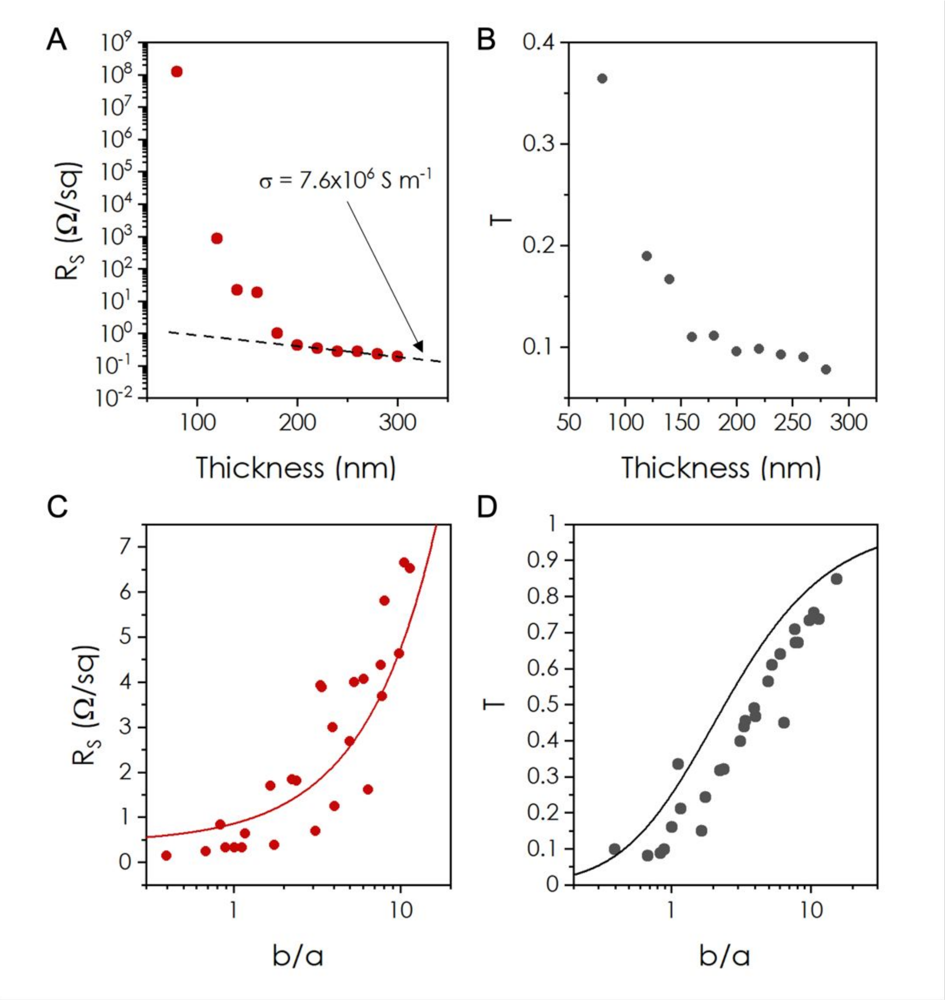

Figure S1: **Inkjet-printed transparent conductors**. The data in (A) and (B) were created by printing continuous networks of AgNS to assess the transmittance of the material itself while (C) and (D) are grids. In (**A**), we find very high R_S for very thin networks but as we move into the thickness-independent conductivity regime, R_S falls off as t^{-1} as expected, with a conductivity of ~ 7.6x10^6 S m^{-1} in line with our previous report. However as shown in (**B**), the transmittance falls to below 10% for networks with the lowest R_S meaning continuous films are completely unsuitable for transparent applications. This motivates the move to grid structures. (**C**) shows R_S vs b/a with a fixed at 1 mm. We find that the data are fit well by Eq. (2) in the main text with $R_{S,Line}$ of 0.43 Ω/sq, consistent with the $R_{S,Line}$ found for the aerosol-jet samples in Figure 3 in the main text. However the data is very scattered owing to the poor resolution of the commercial inkjet printer which makes reproducibility difficult. (**D**) shows the transmittance of the grids vs b/a with a set to 1 mm and the fit to Eq. (1) in the main text.

S2: Estimation of line width and aperture size

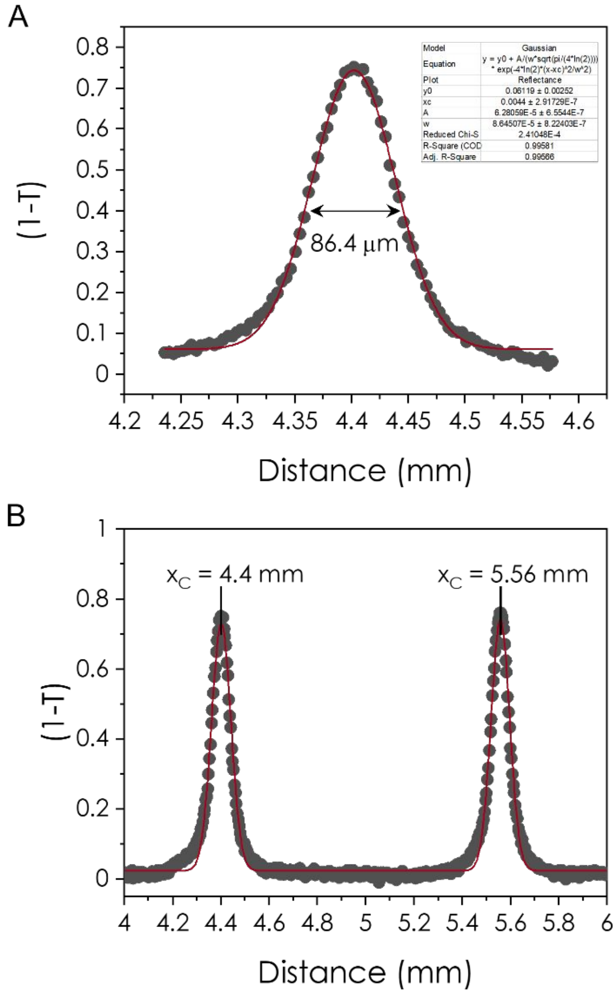

Figure S2: **Estimation of *a* and *b***. Line profiles from the 38 nm grid in Figure 1B in the main text. **A**) The profile of an individual line (the right-hand peak in Figure 1C) fit to a gaussian to extract the FWHM, or line width *a*, which in this case is 84.6 μm. **B**) Fitting these peaks also gives the central position of the peak which allows the aperture size, *b*, to be calculated by subtracting the linewidth from the centre-to-centre distance. For this grid, the aperture size is therefore 1.07 mm.

S3: The onset of sintering at AgNS junctions

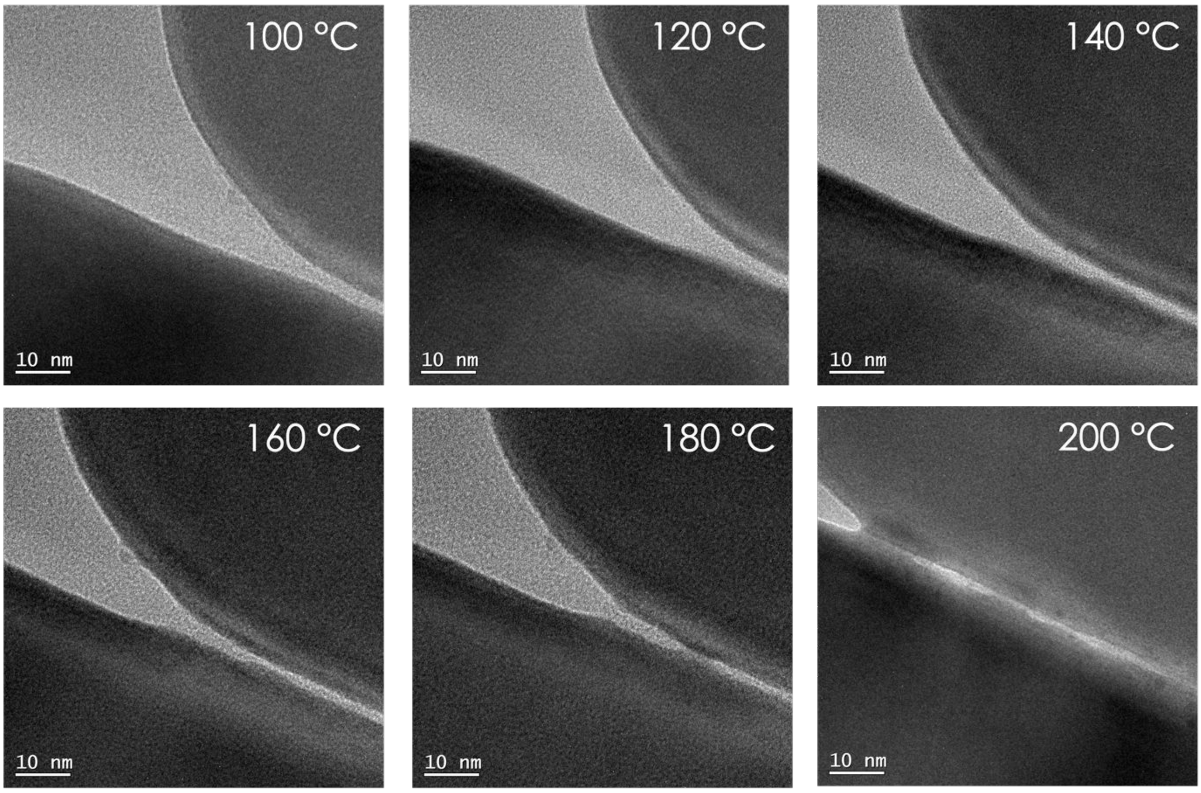

Figure S3: **Onset of sintering between two AgNS with annealing**. Still images from Supplementary Video 1 showing the formation of a weld between the edges of two AgNS to form a large-area junction such as that shown in Figure 2B. The sintering begins at 160 °C with filament formation before the two platelets fully sinter with a dramatic zipper-like effect as shown in Supplementary Video 1.

S4: The formation of filaments across pores

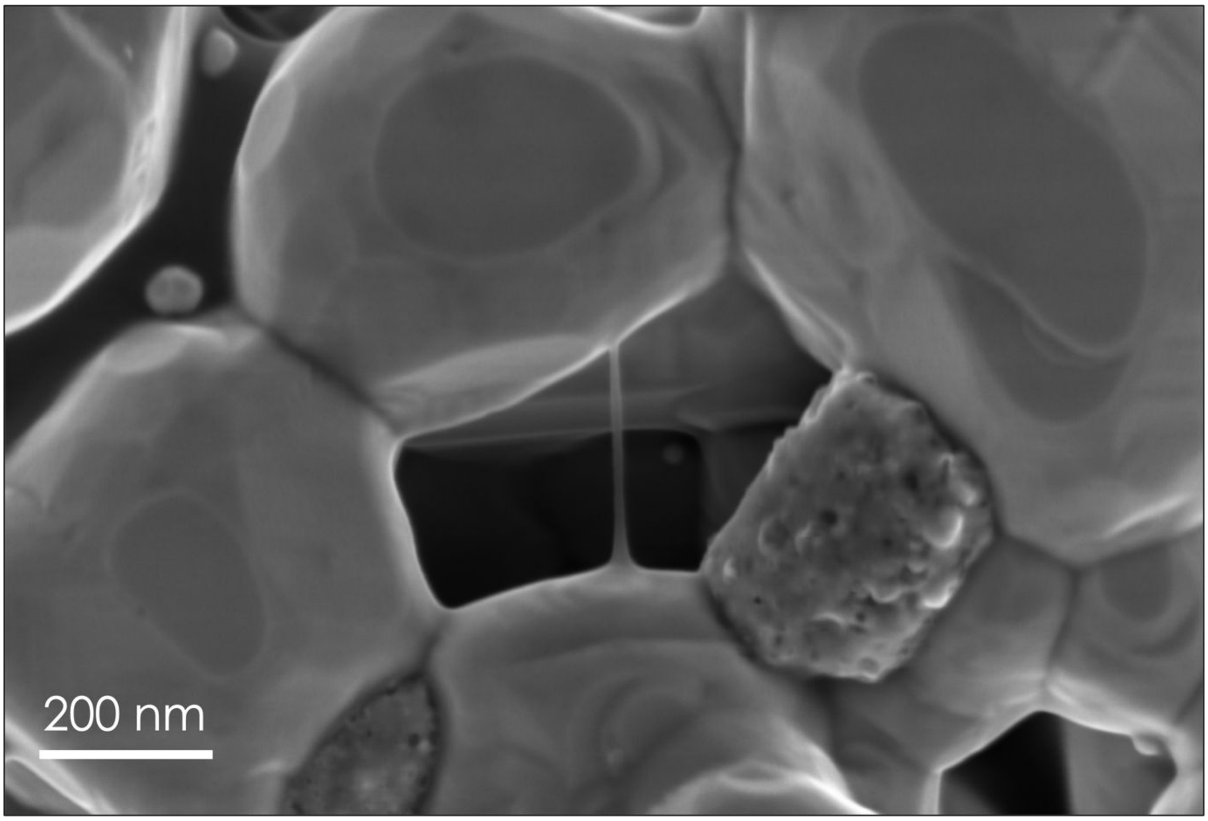

Figure S4: **Filament formation across a network pore**. An SEM image showing a filament between two AgNS bridging a pore ~ 250 nm in distance.

S5: Relationship between transmittance and aperture size

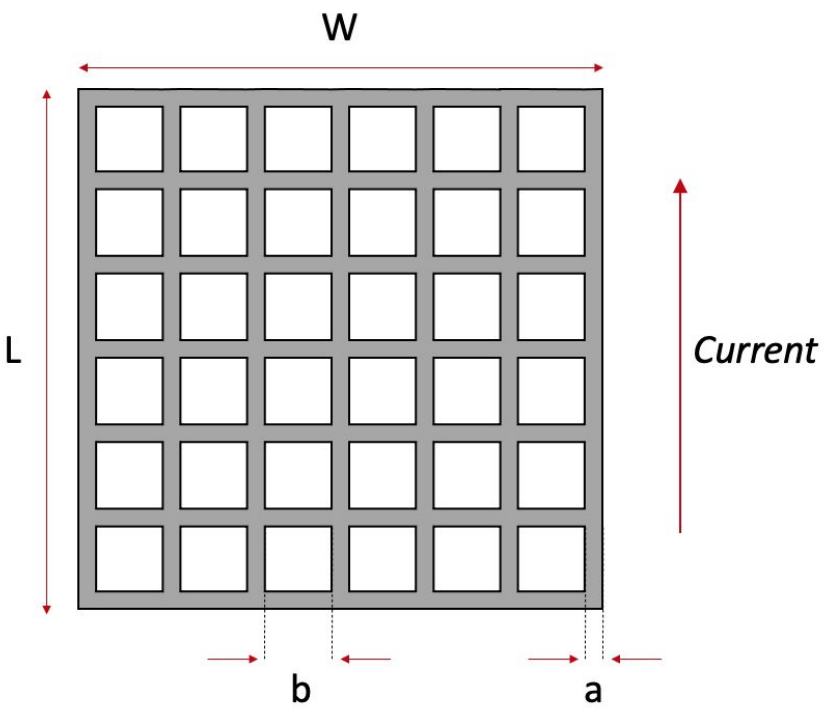

We model the grid as having a length, *L*, a width, *W*, line width, *a*, and aperture size, *b*. We assume the current flows parallel to *L*. We assume the transmittance of the apertures = 1 and the transmittance of the grid lines = 0. We define the fill factor (FF) as the fraction of the grid that is grid lines (or printed material) meaning 1-FF is the fraction of the grid that is apertures. The transmittance is then given by T = (total area of holes) / (total area of grid) =1-FF.

If we assume there are *n* rows and *m* columns of apertures, then the transmittance can be described by T = (number of holes)*(area per hole)/(total area), or

$$T = \frac{nm \times b^2}{L \times W} \qquad (1)$$

The dimensions of the grid can also be described in terms of the line width and apertures as

$$\begin{aligned} L &= nb + (n+1)a \\ W &= mb + (m+1)a \end{aligned} \qquad (2)$$

By combining Eqs. (1) and (2), we find

$$T = \frac{nm \times b^2}{(nb + (n+1)a)(mb + (m+1)a)}$$

$$= \frac{b^2}{(b + (\frac{1+n}{n})a)(b + (\frac{1+m}{m})a)}$$

$$= \frac{1}{(1 + (\frac{1+n}{n})\frac{a}{b})(1 + (\frac{1+m}{m})\frac{a}{b})}$$

$$= \left[\left(1 + (1+1/n)\frac{a}{b}\right)\left(1 + (1+1/m)\frac{a}{b}\right)\right]^{-1}$$

However, if *n* and *m* are very large, then the transmittance can be described as

$$T \approx \left[1 + \frac{1}{b/a}\right]^{-2}$$

S6: Relationship between sheet resistance and aperture size

For current flowing vertically through the grid, the resistance is that associated with m+1 parallel, vertical lines of width *a* and length *L*. As each vertical grid line represents a resistor in parallel, the total resistance of the grid is the sum of the $1/R_{Line}$ values such that

$$\frac{1}{R_{grid}} = (m+1)\frac{1}{R_{line}} \qquad (3)$$

We convert the resistance to sheet resistance using $R_S = R*(W/L)$ for the sheet resistance of the grid, $R_{S,Grid}$ and using $R_S = R*(a/L)$ for the sheet resistance of an individual line, $R_{S,Line}$. Then Eq. (3) becomes

$$\frac{1}{R_{S,grid} L / W} = (m+1)\frac{1}{R_{S,line} L / a}$$

So

$$\frac{W}{R_{S,grid}} = (m+1)\frac{a}{R_{S,line}}$$

Or

$$R_{S,grid} = \frac{R_{S,line} W / a}{(m+1)}$$

Taking $W = mb + (m+1)a$ and assuming that when m is large $W \approx m(a+b)$, this gives

$$R_{S,grid} = \frac{R_{S,line} W / a}{(m+1)} = \frac{R_{S,line} m(a+b) / a}{(m+1)} \approx \frac{R_{S,line}(a+b)}{a}$$

Which, rearranging gives

$$R_{S,grid} \approx R_{S,line}\left(1+\frac{b}{a}\right)$$

Finally, we note that the sheet resistance of a line is related to the line conductivity and thickness by $R_{S,line} = (\sigma_L t)^{-1}$, so

$$R_{S,grid} \approx (\sigma_L t)^{-1}\left(1+\frac{b}{a}\right)$$

S7: Relationship between transmittance and sheet resistance

To relate the transmittance and the sheet resistance to each other, we combine

$$1 - FF = T \approx \left[1 + \frac{1}{b/a}\right]^{-2}$$

with the expression for sheet resistance

$$\frac{b}{a} \approx \frac{R_{S,grid}}{R_{S,line}} - 1$$

which gives

$$1 - FF = T \approx \left[1 + \frac{1}{b/a}\right]^{-2} = \left[1 + \frac{1}{\frac{R_{S,grid}}{R_{S,line}} - 1}\right]^{-2} = \left[1 + \left(\frac{R_{S,grid}}{R_{S,line}} - 1\right)^{-1}\right]^{-2} = \left[1 + \left(R_{S,G}\sigma_L t - 1\right)^{-1}\right]^{-2}$$

S8: Grid and Line Uniformity

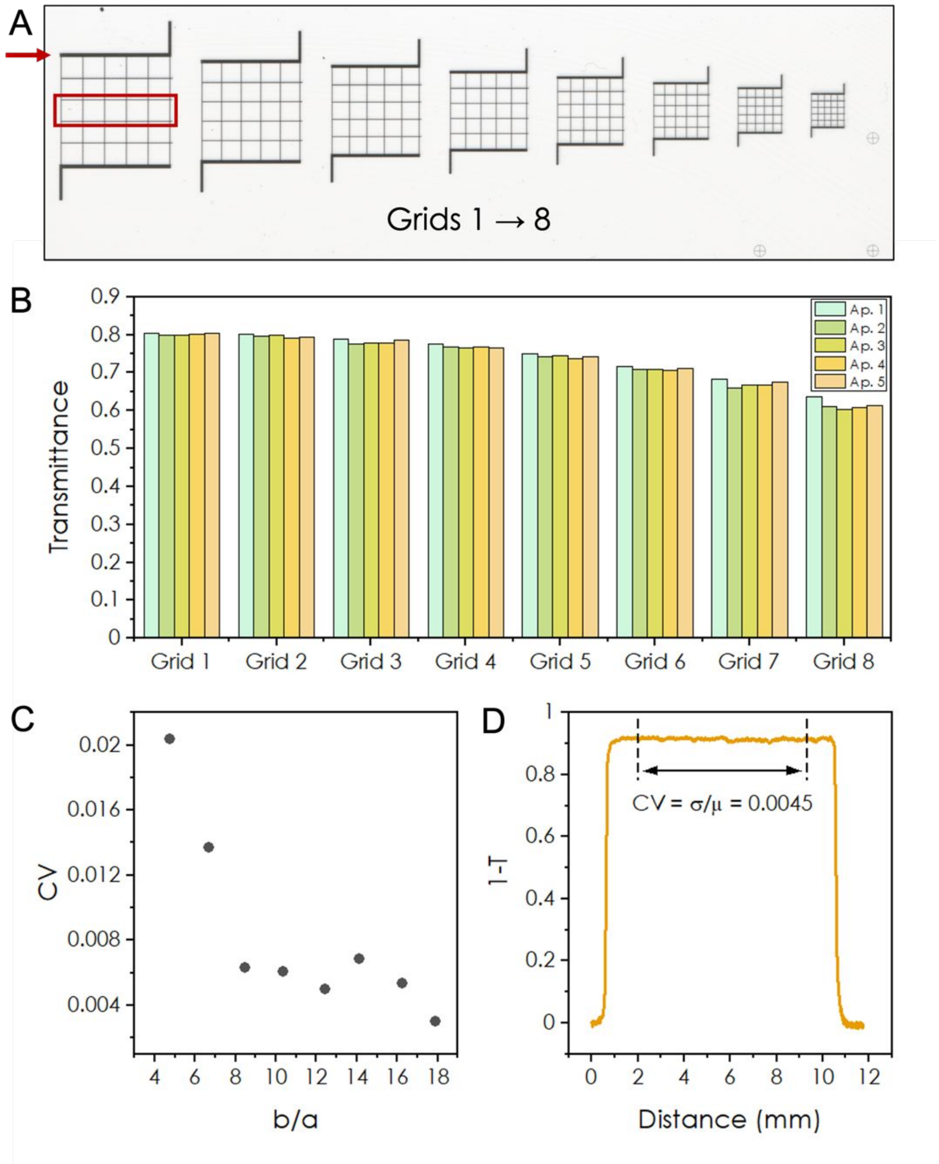

Figure S8: **Assessment of grid and line uniformity**. **A**) A transmission scan of the grids with varied b/a shown in Figure 4A in the main text. **B**) The transmittance of the 5 central apertures for each grid (e.g. those highlighted by the red box in (A) labelled Ap. 1, Ap. 2, etc. from left to right) to assess the uniformity of each aperture. We see a decrease in transmittance with decreasing b/a as expected, however when the aperture size trends towards that of the line width we also find that the transmittance of each aperture becomes more variable. **C**) A plot of the coefficient of variation (CV) for each grid in (B). CV is the ratio of the standard deviation to the mean value and is an indicator of homogeneity across samples. We find while the CV values are very low in general, grids with b/a < 8 show higher values which we attribute to the effect of line edge roughness. **D**) A profile along the line indicated by the red arrow in (A). The printed lines are highly uniform with a low CV of 0.0045 across 7 mm of length.
